# Secondhand Smoke Exposure and Depressive Symptoms Among Nonsmokers in China

**DOI:** 10.1155/bn/1170641

**Published:** 2025-11-19

**Authors:** Xia Cui, Wei Sen Zhang, Lin Xu

**Affiliations:** ^1^ Mental Health Education and Counseling Center, Guangdong Industry Polytechnic University, Guangzhou, China; ^2^ Molecular Epidemiology Research Center, Guangzhou Twelfth People′s Hospital, Guangzhou, China; ^3^ Institute of Applied Health Research, University of Birmingham, Birmingham, UK, birmingham.ac.uk; ^4^ School of Public Health, The University of Hong Kong, Hong Kong, China, hku.hk; ^5^ School of Public Health, Sun Yat-sen University, Guangzhou, China, sysu.edu.cn

## Abstract

**Background:**

Secondhand smoke exposure (SHSE) remains widespread in China and may be linked to mental health outcomes, yet evidence among older adults is limited. We examined the association between SHSE and depressive symptoms in a large cohort of nonsmoking older Chinese adults.

**Methods:**

We conducted a cross‐sectional analysis of 7958 nonsmoking participants aged 50 years or older from the Guangzhou Biobank Cohort Study. SHSE was assessed through structured interviews and quantified as cumulative exposure (in years at 40 h/week) and by context (home and workplace). Depressive symptoms were measured using the 15‐item Geriatric Depression Scale (GDS‐15). Linear regression models were used to examine associations between SHSE and GDS‐15 scores, adjusting for age, sex, education, occupation, income, physical activity, and alcohol use.

**Results:**

Higher cumulative SHSE was associated with greater depressive symptom severity. Participants with more than 5 years of exposure had significantly higher GDS‐15 scores than those with less than 2 years (adjusted *β* 0.21, 95% CI 0.09–0.32; *p* < 0.001). Workplace exposure was independently associated with higher GDS‐15 scores (*β* 0.23, 95% CI 0.08–0.38), while the association for home exposure was weaker and nonsignificant after adjustment. A greater number of smokers and higher frequency of SHSE at home were also linked to elevated GDS‐15 scores. No associations were observed with childhood exposure.

**Conclusion:**

Among older nonsmoking adults, prolonged SHSE, particularly in workplace settings, showed a positive association with depressive symptom severity, although the direction of this association cannot be determined. These cross‐sectional associations warrant investigation in prospective studies to determine whether SHSE exposure precedes depression onset and whether the relationship is causal or reflects shared risk factors.

## 1. Introduction

Globally, depression is one of the leading contributors to disease burden [1]. According to Global Burden of Disease (GBD) 2019 estimates, approximately 280 million individuals were affected, with older age groups showing notably higher prevalence rates [2]. In China, nearly 50 million people are estimated to be living with depression, with evidence indicating an increasing prevalence among older adults [3]. Depression in this demographic is associated not only with diminished quality of life and increased healthcare utilization but also with greater risks of morbidity and mortality [4].

Secondhand smoke exposure (SHSE) remains a significant public health concern [5]. SHSE encompasses involuntary inhalation of mainstream and side‐stream tobacco smoke, and it is recognized as a risk factor for a variety of chronic diseases. Globally, over 1.2 million deaths annually are attributed to SHS, with nearly 300 million nonsmokers in China alone reportedly exposed to SHS, often within household or workplace environments [6].

A growing body of literature suggests a potential association between SHSE and depressive symptoms (DSs) [7–12]. However, most previous studies have concentrated on younger populations, including children [13], adolescents [5, 7, 11, 14], and women of reproductive age [15, 16]. Few studies have explored this association specifically in older adults, despite their elevated vulnerability to both SHSE and depression [17]. Moreover, previous research presents heterogeneous findings, partly due to differences in measurement methods, populations studied, and confounder control [9, 10]. While some studies have identified a dose–response association between SHSE and DS, others have yielded null or inconsistent results, particularly regarding gender‐specific effects and contextual factors such as exposure environment [9, 10].

Given the high prevalence of both SHSE and depression among older Chinese adults and the limitations in existing evidence, we aimed to examine the association between cumulative and contextual SHSE and DS severity, acknowledging the potential for bidirectional relationships. This hypothesis was informed by previous studies in younger populations and sought to assess whether similar associations are present among older nonsmokers in a large community‐based cohort. By leveraging data from the Guangzhou Biobank Cohort Study (GBCS), this investigation addresses an important gap in the literature and provides evidence that may inform public health policies targeting mental health and tobacco control in aging populations.

## 2. Methods

This cross‐sectional analysis was based on baseline data from the GBCS, an ongoing prospective cohort established to investigate environmental and genetic determinants of chronic disease in older adults. The GBCS was developed in collaboration between Guangzhou Twelfth People′s Hospital, the University of Hong Kong, and the University of Birmingham. Baseline recruitment occurred between September 2003 and January 2008. Details of the cohort have been reported elsewhere [18].

Briefly, eligible participants were permanent residents of Guangzhou aged 50 years or older and members of the Guangzhou Health and Happiness Association for Respecting the Elderly, a community organization supported by the municipal government. Individuals were included if they were able to walk unaided and were not receiving treatment for major illnesses including chemotherapy, radiotherapy, or dialysis. All participants underwent standardized physical examinations and face‐to‐face interviews conducted by trained staff at the Guangzhou Twelfth People′s Hospital. For the current analysis, participants were excluded if they reported current or former smoking or had missing data on exposure or outcome variables. Data were collected using structured, interviewer‐administered questionnaires. Interviewers were trained in standardized protocols by the study coordination team, and periodic quality control checks were conducted to ensure consistency and accuracy in data entry. Participants with missing data on SHSE or 15‐item Geriatric Depression Scale (GDS‐15) scores were excluded from the analysis. As the proportion of missing data was low, with fewer than 3.5% of participants missing exposure or covariate information, and less than 1% missing GDS‐15 outcome data, we did not impute missing data. The final analytic sample comprised 7958 nonsmokers with complete data on all relevant variables.

Details of SHSE have been reported elsewhere [19, 20]. SHSE was assessed via interviewer‐administered questionnaires. For each setting (home and workplace), participants reported the average number of hours per week they were exposed to SHSE and the number of years they had been exposed. When exposure intensity varied across life stages or environments, cumulative SHSE was calculated by summing the product of weekly exposure hours and years of exposure for each period (e.g., 10 *h*/week for 20 years + 40 *h*/week for 5 years = 200 + 200 = 400 h‐years). This total was then divided by 40 to express cumulative SHSE as the equivalent number of years of full‐time (40 h /week) exposure. Cumulative SHSE was calculated by multiplying the number of hours of exposure per week by the number of years of exposure, and then dividing by 40 to standardize the result against a typical 40‐h workweek. This approach allowed us to express exposure as an equivalent number of years of full‐time exposure (i.e., 40 h/week), facilitating meaningful comparison across participants with varying exposure intensity and duration [19]. Participants were categorized into three exposure groups: < 2 years, 2–5 years, and > 5 years. Additional data on SHSE in home and workplace settings were collected and categorized using the same thresholds. DSs were measured using the validated GDS‐15, with scores ranging from 0 to 15. The continuous GDS‐15 score was analyzed [21, 22].

Covariates were selected a priori based on existing evidence. These included age (continuous), sex, educational attainment (primary or below, middle school, and college or above), occupation (manual, nonmanual, and other), personal and household income (categorical), alcohol consumption (never, former, and current), and physical activity level derived from the International Physical Activity Questionnaire (low, moderate, and high).

Descriptive analyses were used to summarize baseline characteristics across SHSE categories. Categorical variables were compared using chi‐squared tests and continuous variables using analysis of variance or Kruskal–Wallis tests, as appropriate. Multivariable linear regression models were used to estimate adjusted associations between SHSE and GDS‐15 scores. Regression coefficient *β* and 95% confidence intervals (CIs) were reported for linear regression models. All analyses were conducted using Stata Version 17. A two‐sided *p* value < 0.05 was considered statistically significant.

Ethical approval for the GBCS was granted by the Guangzhou Medical Ethics Committee of the Chinese Medical Association (No. 200308). Written informed consent was obtained from all participants.

## 3. Results

A total of 7958 nonsmoking participants aged 50 years or older were included in the analysis. Among these, 88.3% were women, and the mean age was 59.6 years (SD 7.4). The distribution of sociodemographic and behavioral factors differed across categories of cumulative SHSE. Higher SHSE was associated with increased prevalence of physical inactivity and current alcohol use, while other covariates such as education, occupation, and income showed less pronounced variation (Table 1).

**Table 1:** Characteristics of nonsmoking older adults by cumulative secondhand smoke exposure.

**Variables**	**Adulthood home and work exposure**			**p** **value**
	**< 2 years of 40 h/week**	**2–5 years of 40 h/week**	**> 5 years of 40 h/week**	
Sex, *n* (%)				0.09
Female	3308 (87.6)	1230 (89.6)	2493 (88.8)	
Male	469 (12.4)	143 (10.4)	315 (11.2)	
Age (years), mean (SD)	60.4 (7.8)	59 (7.1)	59.6 (7.3)	< 0.001
Education, *n* (%)				0.02
Primary or below	1479 (39.2)	481 (35)	1022 (36.4)	
Middle school	1984 (52.5)	780 (56.8)	1566 (55.8)	
College or above	314 (8.3)	112 (8.2)	220 (7.8)	
Occupation, *n* (%)				0.009
Manual	2419 (65.1)	844 (62.5)	1745 (62.9)	
Nonmanual	723 (19.5)	257 (19)	514 (18.5)	
Other	572 (15.4)	249 (18.4)	515 (18.6)	
Personal annual income (RMB), *n* (%)				< 0.001
< 5000	528 (14)	156 (11.4)	297 (10.6)	
5000	575 (15.2)	183 (13.3)	368 (13.1)	
10,000	1714 (45.4)	689 (50.2)	1417 (50.5)	
15,000	432 (11.4)	170 (12.4)	377 (13.4)	
20,000	195 (5.2)	71 (5.2)	153 (5.5)	
≥ 30,000	146 (3.9)	45 (3.3)	91 (3.2)	
Do not know	185 (4.9)	59 (4.3)	103 (3.7)	
Annual household income (RMB), *n* (%)				0.07
< 5000	85 (2.3)	22 (1.6)	45 (1.6)	
5000	99 (2.6)	34 (2.5)	80 (2.9)	
10,000	487 (12.9)	168 (12.2)	322 (11.5)	
20,000	689 (18.3)	240 (17.5)	481 (17.1)	
30,000	935 (24.8)	397 (28.9)	724 (25.8)	
≥ 50,000	857 (22.7)	307 (22.4)	685 (24.4)	
Do not know	623 (16.5)	205 (14.9)	469 (16.7)	
IPAQ physical activity, *n* (%)				< 0.001
Inactive	220 (5.8)	82 (6)	317 (11.3)	
Moderate	899 (23.8)	317 (23.1)	872 (31.1)	
Active	2658 (70.4)	974 (70.9)	1619 (57.7)	
Alcohol drinking, *n* (%)				< 0.001
Never	2267 (60.4)	736 (54.2)	1601 (57.4)	
Former	131 (3.5)	77 (5.7)	171 (6.1)	
Current	1354 (36.1)	545 (40.1)	1018 (36.5)	
GDS‐15 score, median (IQR)	2 (1–3)	2 (1–3)	2 (1–4)	< 0.001

Abbreviations: GDS, geriatric depressive scale; IPAQ, International Physical Activity Questionnaire; IQR, interquartile range; SD, standard deviation.

In adjusted linear regression models, participants with more than 5 years of cumulative SHSE had significantly higher GDS‐15 scores compared with those with less than 2 years of exposure (*β* 0.21, 95% CI 0.09–0.32; *p* < 0.001). No significant association was observed for the 2–5 year exposure group (*β* 0.02, 95% CI –0.12 to 0.16; Table 2). When stratified by exposure context, SHSE at the workplace for more than 5 years was associated with increased GDS‐15 scores (*β* 0.23, 95% CI 0.08–0.38; *p* = 0.004), whereas the association for home exposure, although positive, was not statistically significant (*β* 0.17, 95% CI 0.02–0.32; *p* = 0.06; Table 2).

**Table 2:** Adjusted associations between secondhand smoke exposure and depressive symptom scores.

**Variables**	**SHS exposure** ^**a**^			**p** **for trend**
	**< 2 years of 40 h/week**	**2–5 years of 40 h/week**	**> 5 years of 40 h/week**	
Home exposure				
Unadjusted *β* (95% CI)	−0.07 (−0.21 to 0.08)	0.01 (−0.17 to 0.19)	0.32 (0.18 to 0.47) ^∗∗∗^	< 0.001
Adjusted *β* (95% CI)^b^	−0.11 (−0.26 to 0.04)	−0.04 (−0.22 to 0.14)	0.17 (0.02 to 0.32) ^∗∗∗^	0.06
Work exposure				
Unadjusted *β* (95% CI)	−0.06 (−0.2 to 0.08)	−0.05 (−0.22 to 0.12)	0.09 (−0.06 to 0.23)	0.37
Adjusted *β* (95% CI)^b^	0.05 (−0.09 to 0.19)	0.1 (−0.08 to 0.27)	0.23 (0.08 to 0.38) ^∗∗^	0.004
Home and work exposure				
Unadjusted *β* (95% CI)	—	−0.04 (−0.18 to 0.1)	0.20 (0.09 to 0.31) ^∗∗^	0.001
Adjusted *β* (95% CI)^b^	—	0.02 (−0.12 to 0.16)	0.21 (0.09 to 0.32) ^∗∗^	< 0.001

^a^Never exposure to SHS as reference group.

^b^Adjusted for age, sex, occupation, education, income, physical activity, and alcohol drinking.

^∗∗^ *p* < 0.01, and  ^∗∗∗^ *p* < 0.001.

Additional analysis indicated a higher burden of DSs among individuals cohabiting with two or more smokers at home (*β* 0.27, 95% CI 0.03–0.50) and those working in environments with three or more smokers (*β* 0.15, 95% CI 0.03–0.26; Table 3). A dose–response trend was observed between the frequency of SHSE at home and GDS‐15 scores. Daily exposure was associated with an increase in DS score (*β* 0.26, 95% CI 0.05–0.46), with similar elevations noted for exposure one to two times per week (*β* 0.38, 95% CI 0.06–0.69; Table 3). No significant associations were found between SHSE during childhood and DSs in later life.

**Table 3:** Contextual and historical secondhand smoke exposure and depressive symptom scores.

**Variables**	**Unadjusted** **β** **(95% CI)**	**Adjusted** **β** **(95% CI)** ^**a**^
Childhood home smokers		
None	Ref (0.0)	Ref (0.0)
1 person	−0.01 (−0.12 to 0.1)	0.08 (−0.03 to 0.19)
2 people	0.01 (−0.15 to 0.17)	0.13 (−0.03 to 0.29)
Number of smokers in adulthood workplace exposure		
None	Ref (0.0)	Ref (0.0)
1 smoker	−0.04 (−0.28 to 0.2)	0.15 (−0.1 to 0.39)
2 smokers	−0.11 (−0.3 to 0.09)	0.07 (−0.13 to 0.27)
≥ 3 smokers	0.03 (−0.08 to 0.15)	0.15 (0.03 to 0.26) ^∗^
Number of smokers in adulthood home exposure		
None	Ref (0.0)	Ref (0.0)
1 smoker	0.07 (−0.05 to 0.18)	−0.01 (−0.13 to 0.11)
≥ 2 smokers	0.41 (0.18 to 0.64) ^∗∗^	0.27 (0.03 to 0.5) ^∗^
Frequency of exposure to smoke at home		
Never	Ref (0.0)	Ref (0.0)
< 1 times/week	0.21 (−0.16 to 0.57)	0.18 (−0.18 to 0.54)
1–2 times/week	0.28 (−0.04 to 0.59)	0.38 (0.06 to 0.69)
3–4 times/week	0.18 (−0.21 to 0.56)	0.19 (−0.2 to 0.59)
5–6 times/week	0.58 (−0.12 to 1.28)	0.6 (−0.09 to 1.29)
Daily	0.25 (0.05 to 0.46) ^∗^	0.26 (0.05 to 0.46) ^∗^

^a^Adjusted for age, sex, occupation, education, income, physical activity, and alcohol drinking.

^∗^ *p* < 0.05 and  ^∗∗^ *p* < 0.01.

We tested for interactions between SHSE and sex, socioeconomic status, and health status and found no significant effect modification (*p* for interactions from 0.32 to 0.68). Therefore, all analyses were conducted in the total population. As multiple SHSE exposure variables were analyzed, we considered the potential for inflated Type I error. Although analyses were hypothesis‐driven and prespecified, we examined the robustness of findings under conservative correction methods. Using a Bonferroni correction for the number of primary SHSE metrics assessed (*n* = 6), the corrected significance threshold would be *p* < 0.008. The main associations between cumulative and workplace SHSE with DSs (both *p* < 0.005) remained statistically significant under this threshold, suggesting key findings are unlikely to be due to chance.

## 4. Discussion

In this large, community‐based study of older nonsmoking Chinese adults, higher levels of SHSE were positively associated with DSs. After adjusting for sociodemographic and behavioral factors, participants with more than 5 years of cumulative SHSE had significantly higher GDS‐15 scores than those with less than 2 years of exposure. A similar association was observed for SHSE in the workplace, whereas home‐based exposure showed a weaker and nonsignificant effect after adjustment. A dose–response relationship was evident for the number of smokers in both the home and workplace and for increasing the frequency of exposure at home. These findings were consistent across multiple measures of exposure and robust in multivariable models. The observed associations underscore the mental health burden attributable to SHSE and highlight the potential for smoke‐free policies to serve as a modifiable intervention target for improving psychological well‐being in aging populations. However, as this study is cross‐sectional, we cannot establish the directionality of the observed associations. It is possible that individuals with DSs may be more likely to perceive or report exposure to SHS, or that shared underlying factors, such as lower socioeconomic status or occupational environments, may contribute to both higher SHSE and elevated DSs. Therefore, our findings should be interpreted as associative rather than causal.

Although the observed effect sizes were small in absolute terms (e.g., *β* = 0.21 on a 15‐point scale), and likely not clinically meaningful at the individual level, the association remained statistically significant. Further research is needed to determine whether such small differences have cumulative or long‐term relevance in population mental health. The absence of an association between childhood SHSE and late‐life DSs may reflect recall error, weaker long‐term effects of early exposure, or limited variability in childhood SHSE reports within this cohort. Our results are consistent with several studies and meta‐analyses reporting a positive association between SHSE and DSs [9, 10], although the broader literature remains mixed, with some studies reporting null or inconsistent findings [23, 24]. This heterogeneity may reflect differences in population characteristics, study design, exposure assessment, or control for confounding. Meta‐analyses have estimated that SHSE is associated with a 32%–60% increased risk of DSs, although these estimates primarily derive from younger populations and special subgroups such as adolescents and pregnant women [9, 10]. The current study extends this evidence to an older adult population in China, a group in which both SHSE and depression remain prevalent and underaddressed.

The dose–response pattern observed, whereby greater cumulative exposure was associated with greater DSs, supports findings from earlier longitudinal and cross‐sectional studies in China [17] and other countries [10, 12, 17, 23, 25]. Moreover, our results reinforce evidence suggesting that the context of exposure may influence outcomes. Similar to previous studies, SHSE in workplace settings had a stronger association with DSs than home‐based exposure, potentially due to differences in exposure intensity or duration. Although our models adjusted for occupational category, the stronger association observed for workplace SHSE may still reflect residual confounding from unmeasured occupational stressors. Workplace SHSE may act as a proxy for adverse employment conditions, such as poor working environments, low job control, or job dissatisfaction, which are known to be associated with depression. This potential for residual confounding limits the extent to which a direct effect of SHSE can be inferred. Additionally, the absence of a clear dose–response relationship across household smoking categories, whereby exposure to one household smoker was not associated with DSs, but exposure to two or more smokers was, undermines a causal interpretation. This pattern may reflect unmeasured household‐level confounders, such as housing density, socioeconomic disadvantage, family stress, or other shared environmental and behavioral characteristics, rather than a true biological gradient of SHSE.

Our findings regarding gender differences are partially consistent with existing literature [26, 27]. Women were found to have a higher risk of DSs, which aligns with global trends. However, we did not find a statistically significant interaction between sex and SHSE in relation to DS, mirroring results from some prior studies [9] but contrasting with others that reported stronger associations in women [26]. The inconsistency in findings may reflect differing methodologies, cultural contexts, and definitions of SHSE.

Several plausible mechanisms have been proposed to explain associations between SHSE and DSs, although the current study cannot establish whether SHSE contributes causally to these outcomes. SHSE has been shown to induce systemic inflammation [28], oxidative stress [29], and neurochemical alterations [30, 31], including reduced dopamine availability, all of which are implicated in the pathophysiology of depression. Additionally, SHSE may be a proxy for lower socioeconomic status [32], adverse work environments [33], and chronic psychosocial stress [34], which are independently associated with depressive outcomes. These social and environmental factors could help explain the link with DSs.

A major strength of this study is its large, well‐characterized sample derived from a population‐based cohort. The use of a validated depression screening tool (GDS‐15) and comprehensive adjustment for confounding variables enhances the reliability of the results. Moreover, our stratified analysis by exposure context adds nuance to the interpretation of findings. However, several limitations must be acknowledged. The cross‐sectional design precludes causal inference, and the potential for reverse causality cannot be excluded. All exposure data were self‐reported, which may introduce recall or social desirability bias. Another key limitation is the reliance on self‐reported SHSE, which introduces the potential for recall bias, particularly in the retrospective estimation of cumulative exposure over long periods. Participants with current DSs may be more inclined to perceive or recall past exposures as more intense or frequent, introducing the possibility of differential misclassification. We did not perform formal sensitivity analyses to test the robustness of findings under different assumptions about residual confounding or exposure misclassification. This represents a limitation of the current analysis and should be addressed in future studies with more detailed exposure and longitudinal data. Moreover, underreporting by individuals with less awareness or concern about SHS could result in nondifferential misclassification, potentially biasing results toward the null. Unfortunately, the absence of biomarker validation (e.g., serum or urinary cotinine) precludes us from quantitatively assessing the extent or direction of this bias. Objective biomarkers such as serum cotinine would have strengthened exposure assessment. Furthermore, participants were recruited from a community‐based organization and were required to be ambulatory, which may limit the generalizability of findings to the broader older adult population in China, though the internal validity of the observed associations is unlikely to be affected. Given that the sample was predominantly female, the study may have been underpowered to detect sex‐specific associations, and the findings may not be fully generalizable to older men. Furthermore, the study may also have been underpowered to test the interactions by socioeconomic status or health status due to small subgroup sizes. Although internal validity is likely preserved through adjustment for sociodemographic and health covariates, selection factors related to gender, health status, and social engagement may have attenuated or amplified the observed associations in unpredictable ways. Finally, unmeasured confounders, such as environmental pollutants, chronic disease burden, mental health comorbidities, medication use, and social support, may have influenced the observed associations.

Given the high prevalence of SHSE in China and its association with DSs observed in this study, tobacco control policies, including comprehensive smoking bans in workplaces and public settings, may have benefits extending beyond physical health. These measures could contribute to the reduction of mental health burdens in aging populations.

In conclusion, prolonged SHSE was associated with greater severity of DSs among older nonsmoking Chinese adults, particularly with cumulative and workplace exposure. These cross‐sectional associations warrant investigation in prospective studies to determine whether SHSE exposure precedes depression onset and whether the relationship is causal or reflects shared risk factors.

## Ethics Statement

The study was conducted according to the guidelines of the Declaration of Helsinki and approved by the Guangzhou Medical Ethics Committee of the Chinese Medical Association. Informed consent was obtained from all participants involved in the study.

## Consent

The authors have nothing to report.

## Disclosure

All authors read and approved the final manuscript.

## Conflicts of Interest

The authors declare no conflicts of interest.

## Author Contributions

X.C., L.X., and W.S.Z. made substantial contributions to the conception and design, acquisition of funding, and interpretation of data. X.C. and L.X. analyzed the data. X.C. and L.X. drafted the article. L.X. and W.S.Z. revised it critically for important intellectual content.

## Funding

This study was funded by the National Natural Science Foundation of China (10.13039/501100001809) (82373661).

## Data Availability

Due to ethical restrictions protecting patient privacy, data is available upon request from the Guangzhou Biobank Cohort Study Data Access Committee. Please contact us at gbcsdata@hku.hk for fielding data access requests.
